# Dengue Virus-2 Infection Affects Fecundity and Elicits Specific Transcriptional Changes in the Ovaries of *Aedes aegypti* Mosquitoes

**DOI:** 10.3389/fmicb.2022.886787

**Published:** 2022-06-23

**Authors:** Fabiana Feitosa-Suntheimer, Zheng Zhu, Enzo Mameli, Gargi Dayama, Alexander S. Gold, Aditi Broos-Caldwell, Andrea Troupin, Meagan Rippee-Brooks, Ronald B. Corley, Nelson C. Lau, Tonya M. Colpitts, Berlin Londoño-Renteria

**Affiliations:** ^1^Department of Microbiology, Boston University School of Medicine, Boston, MA, United States; ^2^National Emerging Infectious Diseases Laboratories, Boston University, Boston, MA, United States; ^3^Department of Biochemistry, Boston University School of Medicine, Boston, MA, United States; ^4^Department of Genetics, Harvard Medical School, Blavatnik Institute, Boston, MA, United States; ^5^School of Public Health and Tropical Medicine, Tulane University, New Orleans, LA, United States; ^6^Department of Biology, Missouri State University, Springfield, MO, United States; ^7^Genome Science Institute, Boston University, Boston, MA, United States; ^8^Department of Entomology, Kansas State University, Manhattan, KS, United States

**Keywords:** *Aedes aegypti*, dengue, host-pathogen interaction, gene expression, mosquito fecundity

## Abstract

Dengue fever (DF), caused by the dengue virus (DENV), is the most burdensome arboviral disease in the world, with an estimated 400 million infections each year. The *Aedes aegypti* mosquito is the main vector of DENV and transmits several other human pathogens, including Zika, yellow fever, and chikungunya viruses. Previous studies have shown that the pathogen infection of mosquitoes can alter reproductive fitness, revealing specific vector-pathogen interactions that are key determinants of vector competence. However, only a handful of studies have examined the effect of DENV infection in *A. aegypti*, showing a reduction in lifespan and fecundity over multiple blood meals. To provide a more comprehensive analysis of the impact of DENV infection on egg laying and fecundity, we assessed egg laying timing in DENV-2 blood-fed mosquitoes (infected group) compared to mock blood-fed mosquitoes (control group). We confirmed a significant decrease in fecundity during the first gonadotrophic cycle. To further investigate this phenotype and the underlying DENV-2 infection-dependent changes in gene expression, we conducted a transcriptomic analysis for differentially expressed genes in the ovaries of *A. aegypti* infected with DENV-2 vs. mock-infected mosquitoes. This analysis reveals several DENV-2-regulated genes; among them, we identified a group of 12 metabolic genes that we validated using reverse transcription-quantitative PCR (RT-qPCR). Interestingly, two genes found to be upregulated in DENV-infected mosquito ovaries exhibited an antiviral role for DENV-2 in an *Aedes* cell line. Altogether, this study offers useful insights into the virus-vector interface, highlighting the importance of gene expression changes in the mosquito’s ovary during DENV-2 infection in the first gonadotrophic  cycle,  triggering  antiviral  responses  that  may  possibly  interfere  with mosquito reproduction. This information is extremely relevant for further investigation of *A. aegypti’s* ability to tolerate viruses since virally infected mosquitoes in nature constitute a powerful source of supporting viruses during intra-epidemic periods, causing a huge burden on the public health system.

## Graphical Abstract

Aedes aegypti first gonadotrophic cycle is altered by DENV-2 infection. 
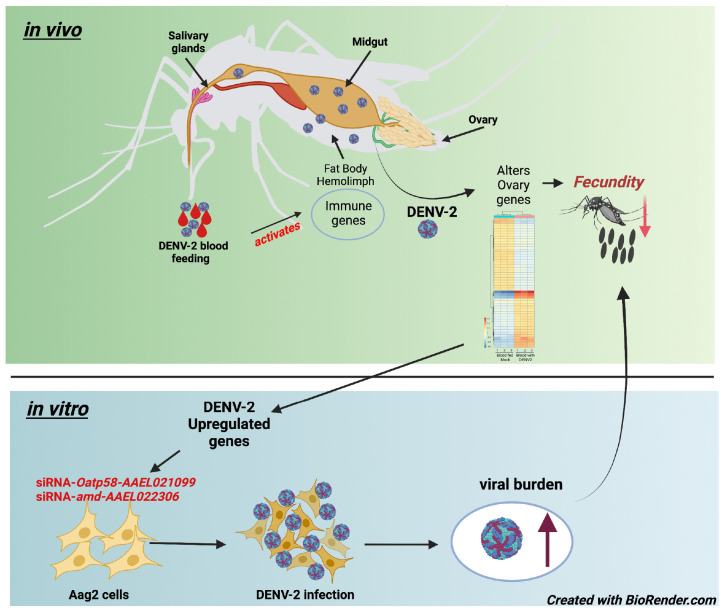

## Introduction

Among emerging and pandemic viruses posing a global threat, dengue fever (DF), caused by dengue virus (DENV), is the most prominent arboviral disease in the world. The World Health Organization (WHO) reports an estimated 100–400 million dengue infections per year worldwide (WHO, 2022) with the largest number of cases reported in 2019. The U.S. Centers for Disease Control and Prevention (CDC) also notes that 40% of the world’s population is at risk for DF (CDC, 2021). Dengue virus serotypes 1–4 (DENV 1–4) are the etiologic agents of dengue fever that lack effective therapies because of ineffective attempts at vaccine development (Izmirly et al., 2020). For example, the only available FDA-approved dengue vaccine, Dengvaxia^®^, has major restrictions on large-scale administration in afflicted countries where dengue fever is endemic. This is due to the problematic side effect of antibody-dependent enhancement (ADE) in a vaccinated patient being previously infected with a DENV serotype different from that of the vaccine antigen (Halstead, 2016).

Research efforts are still needed to better understand how *Aedes aegypti* serves as the main vector of DENV and several other arboviruses, including yellow fever virus (YFV), Zika virus (ZIKV), and chikungunya virus (CHIKV). Although *A. aegypti’s* natural habitats are restricted to tropical and temperate areas, climate change and economic development have increased the density of human populations in the geographic range of mosquitoes, where feeding on humans contributes to expanding mosquito populations and an increase in vector-borne disease cases (Weaver and Barrett, 2004). For example, a recent study suggested a 3.2–4.4% increase in the *A. aegypti* population, and municipal insecticide spraying programs do not represent environmentally sustainable measures to stop the continuing spread of arboviral diseases (Iwamura et al., 2020).

Therefore, there is a relevant need to improve our understanding of how DENV is sustained in mosquitoes after an infectious blood meal as well as investigate its transmission to humans through subsequent infectious mosquito bites. Humans are considered the reservoir for all DENV serotypes (DENV 1–4) in endemic areas of the world, while primates serve as the reservoir for the sylvatic transmission cycle. Furthermore, other authors have suggested that mosquitoes serve as viral reservoirs due to their ability to transmit viruses to vertebrate hosts (Wu et al., 2019). As an example, a study showed that the same DENV serotypes were detected circulating in wild-collected larva and patients’ serum during an outbreak in Sri Lanka (Wijesinghe et al., 2021). After mosquitoes feed on a viremic host, the blood meal is processed in the mosquito midgut, where the virus will infect the midgut epithelial cells and start replicating (Rückert and Ebel, 2018). However, after ingestion of the virus with the bulk of the blood nutrients, the viral particles eventually escape the first barrier of infection, the midgut barrier, to then spread throughout the hemolymph and infect the mosquito hemocytes, fat body, ovaries, and eventually salivary glands (Novelo et al., 2019). After the extrinsic incubation period (EIP) has passed, the infected mosquitoes can then spread arbovirus to several humans through subsequent blood meals (Chan and Johansson, 2012; Carrington and Simmons, 2014; Koh et al., 2018).

Although mosquito somatic tissues, such as the midgut, hemocytes, fat body, and salivary glands, have been previously investigated for their responses to, and capacity for, arbovirus replication (Franz et al., 2015), ovaries have been historically understudied due to the lower viral burden in this tissue. Nevertheless, the viral load in the ovaries is still sufficient for vertical transmission or venereal transmission by infected-born males mating with females (Sánchez-Vargas et al., 2018). The same mechanism of transmission was observed in *A. albopictus* infected with another important flavivirus, ZIKV, where the mosquitoes infected *via* the congenital route could transmit ZIKV to immunocompetent mice (Lai et al., 2020). In field collected *A. aegypti* infected with ZIKV, the vertical transmission was observed as well as the fitness cost of infection in the progeny, showing a reduction in the number of eggs hatched, and slow larvae development (Chaves et al., 2019). Although there is clear evidence of vertical transmission of flaviviruses in *Aedes* mosquitoes, it remains unclear how the mosquito immunity responds to viral infection and what the cost of infection is in mosquito reproduction. The combined events of vertical and venereal transmission are also believed to maintain the virus circulating in wild mosquitoes. Thus, the importance of mosquito ovaries as a site of viral replication, as well as the possible link between activation of mosquito innate immune response in this tissue and mosquito reproduction, warrant further investigation.

The mosquito gonadotrophic cycle is well synchronized starting 1 h after blood feeding with the activation of the 20-hydroxyecdysone (20E) pathway and production of yolk protein precursors (YPPs) by the fat body (Hansen et al., 2014). At 72 h after blood feeding, eggs are fully developed, and females start to search for sites to lay their eggs, usually on the inner walls of artificial containers with fresh water (Day, 2016). Female mosquitoes require a blood meal to initiate the vitellogenesis process by the fat body, which will lead to the intake of proteins to start egg production in the ovaries (Attardo et al., 2005). Although mosquito blood feeding of humans is the key route of pathogenic vectoring capacity and a direct need for mosquito reproduction, the effect of viral infection on mosquito ovary function is poorly investigated. To explore the hypothesis that mosquito ovaries are an important tissue intersecting viral replication and mosquito reproduction, we first conducted a study to investigate the impact of DENV-2 infection on the fecundity of *A. aegypti* females. During the first gonadotrophic cycle, we observed a significant reduction in the number of eggs from DENV-infected mosquitoes compared to uninfected counterparts. We then investigated the changes in the ovary transcriptome of *A. aegypti* 3 days after receiving a DENV-2 blood meal (infected group) compared to mock blood-fed mosquitoes (control group). We highlight several genes with physiological and metabolic functions in the ovary that are changing in expression levels during DENV infection. Some DGEs from the mosquito ovary analysis also appeared to be differentially expressed in the mosquito *A. aegypti* Aag2 cell line when subjected to DENV infection. Interestingly, two genes found to be upregulated in DENV-infected mosquito ovaries exhibited an antiviral role for DENV-2 in an *Aedes* cell line. Overall, our study shows that despite the mosquito’s high tolerance to DENV-2 infection *via* blood meal, the ovaries undergo physiological responses to DENV-2 infection as early as 3 days after receiving an infectious blood meal, as reflected by specific transcriptome changes and a reduced fecundity rate of egg laying. Finally, this study set the basis for further investigation of mosquito genes involved in the specific response to DENV infection. These genes possibly orchestrate the delicate balance between immunity and reproduction and contribute to sustaining vector competence. New insights into mosquito tolerance factors could offer new opportunities for vector control strategies aimed at interrupting viral transmission.

## Materials and Methods

### Mosquitoes, DENV-2 Infection, and Blood Feeding

*Aedes aegypti* colony (Rockefeller strain) was obtained from Tulane University. Mosquitoes were maintained in rearing chambers in a secure insectary (arthropod containment level 3) ACL-3 in the NEIDL, following rearing conditions as described by Araujo et al. (2020).

For the transcriptome experiments, human blood from a donor was used (The Blood Center, New Orleans, LA, United States). Briefly, blood in EDTA was centrifuged, and plasma was separated and inactivated at 56^°^C for 1 h. Red blood cells (RBCs) were washed three times with 0.01 M PBS and reconstituted with autologous plasma. For the infection, the blood mixture was then added in a 1:1 ratio to the supernatant from either the DENV-2 culture or uninfected cells. For the feeding experiments, 7–10 day old female *A. aegypti* mosquitoes were starved for 24 h before the feeding. Mosquitoes were fed for 30 min. Engorged females were sorted and kept in the incubator for 3 days with a 10% sucrose solution provided on day 3 after feeding, ovaries from all live mosquitoes were dissected in 0.01M PBS, and RNA was extracted as described below. Midguts were tested for the presence of infection with DENV using reverse transcription-quantitative PCR (RT-qPCR).

For RT-qPCR validation of genes and egg laying, pre-mated females at 7 days old were transferred to individual cups and fasted for 24 h prior to the DENV-2 blood meal or mock blood meal. Females were infected *via* feeding on a Hemotek Feeding System, and an infectious blood meal composed of mice blood and DENV-2 (2.7 × 10^7^ ffu/ml) supernatant at a 1:1 dilution was given to the mosquitoes (infected group). For the mock blood feeding (control group), females were fed with clean blood composed of Vero-E6 cell supernatant and mouse blood at a 1:1 dilution. The blood was freshly collected from Balb/C mice through cardiac puncture and placed in a heparin-coated tube according to standard procedures approved by IACUC PROTO201900005. The whole blood was centrifuged at 3,000 rpm for 10 min to separate plasma and RBCs. RBCs were washed three times in 0.01M PBS to remove anticoagulants, and plasma was heat-inactivated at 56^°^C for 1 h. RBCs were resuspended with plasma and used immediately for mosquito blood feedings. Mosquitoes were allowed to feed for 30 min. Engorged females from each group were sorted into cups and provided with a 10% sucrose solution and returned to the incubator. Ovaries from females of each group were dissected in 0.01M PBS 3 days after blood feeding. Carcasses from the DENV-infected group were tested for DENV-2 using RT-qPCR, and a pool with the corresponding positive ovaries was used in the RT-qPCR for validation of the volcano plot genes. Individual egg-laying females were placed in individual tubes with 10% sucrose provided. Eggs were counted daily, from 3 to 7 days after receiving a blood meal.

### Cell Cultures and Virus

The *A. aegypti* embryonic-derived cell line, Aag2 (gift from Dr. Michael J. Conway), was cultivated at 28^°^C in an incubator with 5% CO_2_ in high glucose Dulbecco’s Modified Eagle’s Medium, DMEM (Gibco), supplemented with 10% heat-inactivated fetal bovine serum (FBS) (Gemini), 1% Tryptose Phosphate Broth (Gibco), and Antibiotic-Antimycotic (Gibco). Aag2 cells were used for *in vitro* validation of gene expression from selected genes.

The dengue virus 2, New Guinea C (DENV-2-NGC) strain was obtained from BEI resources. The virus was passaged into Vero-E6 cells and cultured for 5 days in high glucose DMEM (Gibco) medium supplemented with 2% heat-inactivated FBS (Gemini) at 37^°^C in an incubator with 5% CO_2_. After visualization of cytopathic effect (CPE), the viral supernatant was centrifuged, spun down for 10 min at 4,000 rpm, and filtered using a 0.45 (μm) syringe filter. The virus was harvested and aliquoted in cryotubes and stored at -80^°^C freezer to use for the mosquitoes’ infectious blood meal. DENV-2 was also passaged into Vero-E6 cells for focus forming assay (ffa) and the viral titer was obtained.

### RNA Sequencing

A pool of 100 ovaries was dissected in 0.01M PBS from females 3 days post DENV-2 blood feeding (DENV infected) and the 100 ovaries from the control group, 3 days post uninfected blood feeding (mock). This was performed in triplicate, and gene expression of infected ovaries was compared to the uninfected control group. Tissues were homogenized in 100 μl of RNA lysis buffer using a pestle and an additional 500 μl of buffer was added to each sample. Total RNA was extracted from infected and uninfected samples using the Qiagen RNeasy Plus Mini-Kit (Qiagen, 74136) per the manufacturer’s instructions. RNA sequencing was performed from mosquito samples in three biological replicates by outsourcing to Genewiz, which conducted poly(A) selection and standard strand-specific single-end RNA sequencing (RNA-Seq) library construction and sequencing on a NextSeq 500 instrument. A total of six FASTAQ files were generated and deposited at the NIH NCBI Sequence Read Archive (SRA) under the BioProject accession # PRJNA786000.

### Bioinformatics Analysis of RNA Sequencing Data

For quality control, 6 FASTQ files (3 from mock and 3 from DENV infected) were first inspected using FastQC (REF: Andrews S. FastQC: a quality control tool for high throughput sequence data, 2010) to assess any bias due to read quality and length and to confirm the linker adapters had been trimmed. Using the STAR aligner (Dobin A. STAR: ultrafast universal RNA-seq aligner, 2013), RNA-seq reads were then mapped to the reference genome of *A. aegypti* (strain Liverpool AGWG, assembly AaegL5) downloaded from VectorBase (2022), but an additional assignment of Drosophila gene names from FlyBase (Thurmond et al., 2019) was appended to enhance the identification of the mosquito gene’s machine-generated names.

As the standard analysis of DEGs based solely on *p*-values yielded an intractably large list of gene candidates, we modified our approach to filter for robustly affected genes. First, we looked at mosquito gene annotations that have gene ID, such as AAEL###### as described in the mosquito database (VectorBase, 2022) by appending the gene name of the *Drosophila melanogaster* ortholog from FlyBase (Thurmond et al., 2019).

After batch correction and initial filtering in R, overall relatedness can be observed within the mock and DENV-2 infected samples as seen by the clustering in the principal component analysis (PCA) plot (Supplementary Figure 1A). Using estimated read counts, the transcriptomes below the threshold value (est. read count < 1 across all samples) were further filtered using R. Correction was done for batch effects using DESeq2 (Love et al., 2014; Supplementary Figure 1A) for 2 PCAs before and after batch correction. Differential gene expression (DGE) analysis was then performed using DESeq2 as well as EdgeR (Robinson et al., 2009) to generate a robust list of DEGs. In each result set, the most significant DEGs were determined (FDR < 0.05), and a intersect analysis (Venn diagram) showed approximately 91% overlap of DEGs from DESeq2 results (Supplementary Figure 1B). Thus, DESeq2 results were utilized for downstream analysis.

A heatmap was generated to show the top 50 upregulated and downregulated DEGs. Furthermore, the significant DEGs (FDR < 0.05) were filtered for a base mean expression value of 150, and 12 highly expressed genes were picked for validation using RT-qPCR as highlighted in the volcano plot and the heatmap. Gene Ontology (GO) analysis was performed on significant DEGs (FDR < 0.05) using VectorBase’s annotation tool (Ashburner et al., 2000; VectorBase, 2022). The top entries for biological processes, molecular functions, and cellular components, were picked based on an FDR cutoff of 0.005 and excluding GO terms containing 1,000 genes or more to focus on specialized terms.

### Mosquito Fecundity Assay

Mosquito egg laying assays were performed in two biological replicates. Two groups of pre-mated females, 7–10 days old, were used for this assay. DENV-2 infected and mock mosquitoes were blood-fed using a Hemotek Feeding System as previously described. The viral titer used in the infectious blood meal was 2.5 × 10^7^ focus forming units (ffu). Engorged females were sorted into individual 50 ml Falcon tubes for egg laying. Each tube was closed with a piece of mesh cloth and secured with an elastic band. A cotton ball with 10% sucrose was placed on the top of each tube for subsequent feeding of mosquitoes. At approximately 60 h post blood feeding, a strip of wet filter paper was placed inside each tube to allow oviposition. The egg counting started at 3 days post blood feeding and ended at 7 days post blood feeding when females were not laying any more eggs. At the end of the experiment, at 7 days post infection (7 DPI), each mosquito was homogenized for total RNA extraction using the QIAshredder (Qiagen, 79656) and the Qiagen RNeasy Plus Mini-Kit (Qiagen, 74136). Individual mosquitoes were tested for DENV-2 by RT-qPCR using the QuantiFast SYBR Green RT-PCR Kit (Qiagen, 204156). Viral burden from individual mosquitoes was obtained by using a standard curve from an *in vitro* transcription of standard DENV-2 RNA as described below. Only positive females were considered for the egg counting final graph.

### Reverse Transcription-Quantitative PCR Validation of Gene Expression of Selected Genes and Immune Genes

A total of 12 genes were chosen for RT-qPCR validation. At 3 days after infected blood feeding (DENV-2 infected) and uninfected blood feeding (mock), ovaries of 10 females (DENV-2 infected) and ovaries of 10 females (mock) were dissected in 0.01M PBS and homogenized in RLT plus buffer for total RNA extraction per manufacturer’s instructions using the QIAshredder (Qiagen, 79656) and RNeasy Plus Mini Kit (Qiagen, 74136). Individual carcasses of DENV-2 blood-fed mosquitoes were homogenized as described and RNA extracted to be tested for DENV-2, and only the ovaries from positive carcasses were considered for the RT-qPCR assay as described below:

The *in vitro* transcription of standard DENV-2 RNA (ssRNA) was obtained following the methods described by Araujo et al. (2020), using 5 μg of total RNA from DENV-2 NGC infected Vero-E6 cells. The first strand was generated using SuperScript III (Invitrogen, 18080051) with random hexamers, and the cDNA generated was used in PCR reaction with the T7 promoter sequence added to the forward primer DENV2_T7-5′-TAATACGACTCACTATAGGGAGAGCAGATCTCTGATGAA TAACCAACG-3′ and the reverse primer DENV2_Env_RC: 5′-CATTCCAAGTGAGAATCTCTTTGTCA-3′. PCR cycles of 95^°^C for 3 min, followed by 35 repeated cycles of 95^°^C for 30 s, 62^°^C for 30 s, and 72^°^C for 1 min. PCR products were used as a template for the *in vitro* transcription using the MEGAscript T7 Transcription Kit (AMB13345).

Absolute quantitation of viral burden per mosquito was obtained using the RNA from carcasses as a template in the one-step RT-qPCR reaction using the QuantiFast SYBR Green RT-PCR Kit (Qiagen) and a CFX96 Touch Real-Time PCR Detection System (Bio-Rad, California, United States) and calculated using a 10-fold dilution series of DENV2 ssRNA as a standard.

The relative gene expression for all 12 genes selected for validation of RNA-Seq and the immune pathway genes were normalized against the *A. aegypti* actin gene using the 2^–ΔΔ*CT*^ method. Primers used for these assays were designed using the Primer-BLAST tool available online (Ye et al., 2012). All of the primers used in this study can be found in Supplementary Table 1.

### Gene Silencing in Aag2 Cell Line

Transfection of siRNA on Aag2 cells was performed using Lipofectamine 3000 (Thermo Fisher Scientific) according to the manufacturer’s instructions. The siRNA sequences can be found in Supplementary Table 1. Briefly, 5 × 10^4^ cells were seeded into 24-well plates 1 day before transfection. After cells achieved 90% confluency, 10 pmol of gene-targeting siRNA or scrambled siRNA control was mixed with Lipofectamine 3000 (Thermo Fisher Scientific) and Opti-MEM^®^ (Gibco), which was then used to transfect Aag2 cells. At 72 h post transfection, the siRNA-transfected cells were inoculated with DENV-2 at an multiplicity of infection (MOI) of 1 for 2 h at 30°C and 5% CO_2_ incubator. At 2 h post infection (hpi), the inoculum was removed, and the cells were washed once with high glucose DMEM (Gibco). Following washing, DMEM (Gibco) supplemented with 2% FBS was then added to the cells. Time points (cell lysates and viral supernatant) were collected at 24 hpi. Cell lysates were harvested in the RLT buffer by adding beta-mercaptoethanol (β-ME), RNA was extracted using the RNeasy Plus Mini Kit (74106, Qiagen), and RNAi efficiency was validated using qRT-PCR. Cell culture supernatants were collected, and viral titers were obtained by ffa as described. Viral RNA was extracted using the QIAamp Viral RNA Kit (52906, Qiagen). DENV load was then quantified by RT-qPCR using SYBR Green and DENV-2 ssRNA as standard, as we previously described.

### Focus Forming Assay

Following dengue infection, cell supernatants from siRNA-transfected Aag2 cells were collected to measure infection by a focus-forming assay. Vero-E6 cells were seeded in 96-well plates at a density of 10,000 cells per well and cultured in DMEM (Gibco) supplemented with 10% heat-inactivated fetal bovine serum (FBS) (Gemini) and 1% penicillin/streptomycin (Gibco) at 37^°^C in an incubator with 5% CO_2_. At 24 h after seeding, Vero-E6 cells were treated with 100 μl of supernatants from infected cells in a serial 10-fold dilution from undiluted to 10^–7^ diluted in DMEM (Gibco) for 2 h at 37^°^C in an incubator with 5% CO_2_. After this time, the supernatant was removed, the cells were washed with PBS, and 100 μl of overlay consisting of a 1:1 ratio of DMEM:2% FBS media and 5% carboxymethyl cellulose was added to the cells. Infection was allowed to progress for 5 days, after which the overlay was aspirated, and cells were washed with 0.01M PBS, then fixed with 4% paraformaldehyde (Thermo Fisher Scientific) supplemented with 0.1% Triton X-100 (Thermo Fisher Scientific) for 30 min at room temperature. The fixing solution was then aspirated, and cells were washed with 0.01M PBS again, then blocked with 1% bovine serum albumin (BSA) (Millipore Sigma B6917) in 0.01M PBS at room temperature for 1 h. Following this time, the blocking buffer was removed and cells were incubated with flavivirus group antigen-antibody [D1-4G2-4-15 (4G2)] (Novus Biologicals, NBP-52709, Centennial, CO., United States) diluted 1:1,000 in 1% BSA in 0.01M PBS for 2 h at room temperature. After this time, the antibody was removed, cells were washed 3 times with 0.01M PBS, and cells were incubated with IRDye^®^ 680RD Goat anti-Mouse IgG Secondary Antibody (LI-COR^®^ Biosciences, Lincoln, NE, United States) diluted 1:1,000 in 1% BSA in 0.01M PBS for 2 h at room temperature. After this time, the antibody was removed, and cells were washed 3 times with 0.01M PBS. PBS was aspirated and cells were allowed to dry. Focus forming assays were performed in duplicate. Foci were identified by the detection of DENV E-specific signal in the 700 nm channel using the Odyssey CLX300 Near-Infrared Fluorescence Imaging System (LI-COR^®^ Biosciences, Lincoln, NE, United States) and counted and reported as focus forming units (FFU).

## Results

### DENV-2 Reduces Fecundity in *Aedes aegypti* During the First Gonadotrophic Cycle

**FIGURE 1:**
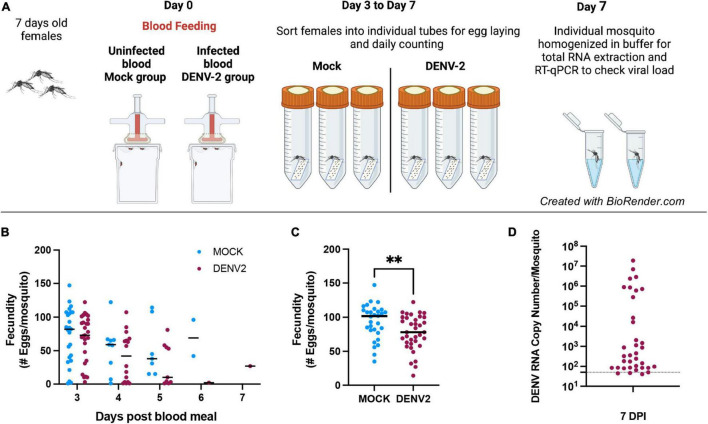
Mosquito egg production can be affected by DENV-2 infection during the first gonadotropic cycle. **(A)** Schematic of the DENV-2 infection process as further detailed in the “Materials and Methods” section. **(B)** Mosquito fecundity after infectious blood meal (DENV2) compared to uninfected blood meal (mock) per time point 3–7 days. **(C)** Overall fecundity of infected mosquitoes. Representative results from two independent experiments analyzed per group ^**^p < 0.005 (Mann-Whitney test). **(D)** Viral load from mosquito bodies at 7 DPI by RT-qPCR using 10-fold dilution of DENV-2 standard RNA, ranging from 10^8^ to 10^1^ copies/μl. Mosquitoes’ body were tested for DENV-2 infection and only positive females were considered for fecundity graphs. Mock *n* = *30*, DENV-2, *n* = *36.*

We aimed to examine the impact of DENV infection on mosquito fecundity. For this, we fed mated 7–10-day old *A. aegypti* females with uninfected blood (mock) vs. blood containing DENV-2 (Figure 1A). We observed that during the first gonadotrophic cycle, the females in the DENV-infected group laid significantly fewer eggs compared to females in the mock blood-fed group. In our fecundity assay, we counted eggs daily from 3–7 days post blood feeding (mock or DENV), and we did not observe significant differences in the timing of oviposition, with most mosquitoes laying most of their eggs 3 days post blood meal (Figure 1B). In the mock group, the average number of eggs laid per female was 93 eggs/female from a total of 30 females analyzed. In the DENV-2 infected group, the average number of eggs laid was 77 eggs/female from a total of 36 females. These data show a reduction of 22.5% in fecundity from the DENV-2-infected mosquitoes (Figure 1C). After egg laying, we confirmed that each female mosquito was infected with DENV using RT-qPCR to detect from 10^1^ to 10^8^ viral copies. The ratio of DENV-2 infection in the mosquitoes was 50% in the first gonadotrophic cycle alone (Figure 1D).

### A Search for Genes Affected in *Aedes aegypti* Ovaries Upon DENV-2 Infection

Since blood feeding triggers changes in complex gene expression in females (Giraldo-Calderón et al., 2020), we wondered if the impact that DENV had on the first gonadotrophic cycle could be linked to an alteration of gene expression profiles during blood feeding. Therefore, we performed RNA-seq and transcriptomic analysis of differentially expressed ovarian genes in *A. aegypti* females at 3 days after DENV-2 blood feeding compared to females at 3 days after uninfected blood feeding (mock). As the standard analysis of DEGs based solely on *p*-values yielded an intractably large list of gene candidates, we modified our approach to filter for robustly affected genes. First, we looked at mosquito gene annotations that have gene IDs, such as AAEL###### as described in the mosquito database (VectorBase, 2022) by appending the gene name of the *Drosophila melanogaster* ortholog from FlyBase (Thurmond et al., 2019).

**FIGURE 2:**
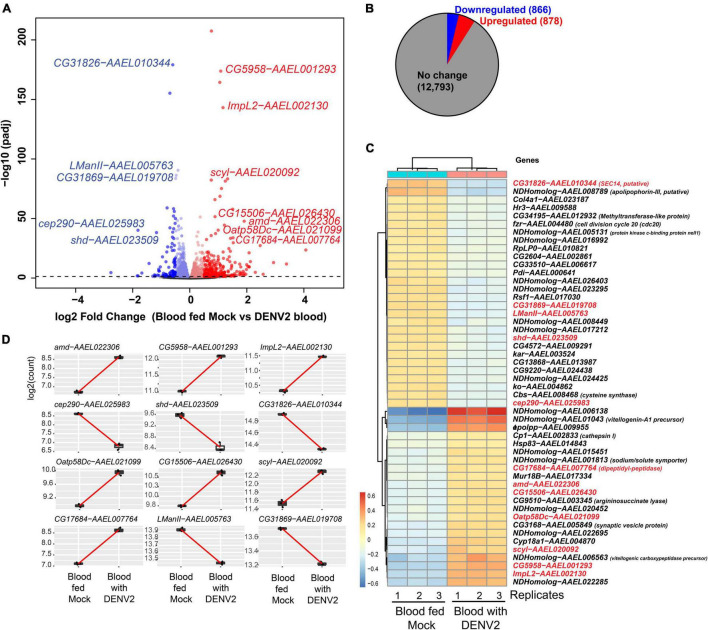
Differential gene expression analysis of *Aedes aegypti* ovaries transcriptomes 3 days after DENV-2 blood feeding vs. mock blood feeding. **(A)** Volcano plot of differentially expressed genes (DEGs) from *A. aegypti* ovaries RNA-seq libraries. **(B)** Fractional proportion of the ovarian DEGs relative to all other *A. aegypti* transcripts. **(C)** Heatmap displaying the top 50 DEGs, and in red text labels are the 12 genes selected for additional downstream studies because the base-mean expression > 200 RPM [or log2(7.6)]. *Drosophila* gene names are shown except for mosquito-specific genes that are named *NDHomolog* because there was no *Drosophila* homologs to be assigned. **(D)** Highlighting the 12 ovarian gene read counts indicate the robustness of the expression change in the read sequencing counts between replicates and the base-mean expression > log2(7.6).

After batch correction and initial filtering in R, overall relatedness can be observed within the mock and DENV-2 infected samples as seen by the clustering in the PCA plot (Supplementary Figure 1A). Using DESeq2 from a total of 14,537 DEGs, we identified that 1,744 (12%) genes were significant at an FDR cutoff of 0.05, of which 878 (6%) were upregulated, while 866 (6%) genes were downregulated in the DENV-2 infected pool (Figures 2A,B). The top 50 DEGs can be observed in the heatmap (Figure 2C). From the volcano plot and heatmap, we chose 12 DEGs for the validation of gene expression by qRT-PCR since this technique is powerful in validating gene expression due to its sensitivity and precision. From the list of 12 genes, seven were upregulated, namely, *amd*-AAEL022306, *CG5958*-AAEL001293, *ImpL2-*AAEL002130, *Oatp58Dc*-AAEL021099, *CG15506-*AAEL026430, *scyl*-AAEL020092, *CG17684-*AAEL007764, and five were downregulated, namely, *cep290-*AAEL025983, *shd*-AAEL023509, *CG31826-*AAEL010344, *LManII*-AAEL005763, and *CG31869-*AAEL019708. These 12 genes (Figure 2D) are shown to be between the most upregulated or most downregulated (basemean > 150), highlighted in red in the heatmap. We also searched for the ortholog using FlyBase to find the predicted function of those 12 genes. The *Drosophila* ortholog gene function of each of the 12 genes can be found in Table 1. The table illustrates the utility of appending the *Drosophila* gene ortholog name to the mosquito gene ID from Vectorbase because 8/12 of these genes lacked a functional description in the 2021 edition of Vectorbase. However, all of these genes had a clear *Drosophila* ortholog for which Flybase contained a rich functional annotation for the gene. A trend of metabolic and physiology-regulation functions is apparent in this list of genes, with functions such as carboxy-lase for *amd-AAEL022306*, membrane transport in *Oatp58Dc-AAEL021099*, dipeptidyl-peptidase in *CG17684-AAEL007764*, and lysosomal alpha-mannosidase in *LMannII-AAEL005763*.

**TABLE 1:** *Aedes aegypti* ovarian genes with differential expression in blood feeding with DENV-2 selected for RT-qPCR validation.

VectorBase gene ID	VectorBase description	*Drosophila* ortholog name	*Drosophila* ortholog gene function
**Unregulated genes selected for validation**			
AAEL022306	Unspecified product	*amd*	A methyl dopa-resistant (amd) encodes a carboxy-lyase involved in catecholamine metabolism, i.e., adrenaline, epinephrine
AAEL002130	Ecdysone inducible protein L2, putative	*lmpL2*	Ecdysone-inducible gene L2. Exhibits insulin binding activity. Involved in several processes, including negative regulation of phosphatidylinositol 3-kinase signaling; positive regulation of entry into reproductive diapause; and regulation of insulin receptor signaling pathway.
AAEL001293	Unspecified product	*CG5958*	Predicted to have phosphatidylinositol bisphosphate binding activity. PIP2 is a minor phospholipid component of cell membranes.
AAEL021099	Unspecified product	*Oatp58Dc*	Organic anion transporting polypeptide 58Dc (Oatp58Dc) encodes a membrane transporter implicated in renal elimination of organic compounds including ouabain and methotrexate. In the perineural glia of the blood brain barrier, the product of Oatp58Dc protects the brain from potentially toxic organic anions in the hemolymph.
AAEL026430	Unspecified product	*CG15506*	No known functions associated, highest express in Drosophila pupation
AAEL020092	Unspecified product	*scyl*	Scylla inhibits cell growth by regulating the Tor pathway upstream of the Tsc1-Tsc2 complex and downstream of Akt1. Acts as cell death activator during head development
AAEL007764	Dipeptidyl-peptidase	*CG17684*	Predicted to have serine-type peptidase activity. Predicted to be involved in proteolysis. Is expressed in adult head and spermatozoon. Human ortholog(s) of this gene implicated in amyotrophic lateral sclerosis; asthma; autosomal dominant non-syndromic intellectual disability 33; and spinal muscular atrophy.
**Downregulated genes selected for validation**			
AAEL023509	Unspecified product	*Shd*	Shade (shd) encodes 20-hydroxylase and is responsible for converting Ecdysone into 20-hydroxyecdysone, the active form of the steroid. It is required in all tissues that produce active Ecdysone and thus contributes to larval moulting, metamorphosis, growth, neuroblast diversity and egg chamber maturation
AAEL025983	Unspecified product	*cep290*	Centrosomal protein 290 kDa (Cep290) encodes a cytoplasmic protein located in the cilium transition zone, which assists in the compartmentalization of the cilium in sensory cilia, and the ciliary tip in sperm flagella. Its roles include coordinated movement behavior and sperm motility
AAEL010344	SEC14, putative	*CG31826*	Predicted to have phosphatidylinositol bisphosphate binding activity. PIP2 is a minor phospholipid component of cell membranes.
AAEL005763	Lysosomal alphaman nosidase	*LManll*	Lysosomal a-mannosidase II (LManll) encodes a mannosyl-oligosaccharide 1,2-alpha-mannosidase involved in the degradation of asparaqine-linked carbohydrates of qlycoproteins.
AAEL019708	Unspecified product	*CG31869*	No known functions associated, widely expressed in many tissues and all stages of *Drosophila.*

### Gene Ontologies of Ovarian DEGs From a DENV-2 Infectious Blood Meal

**FIGURE 3:**
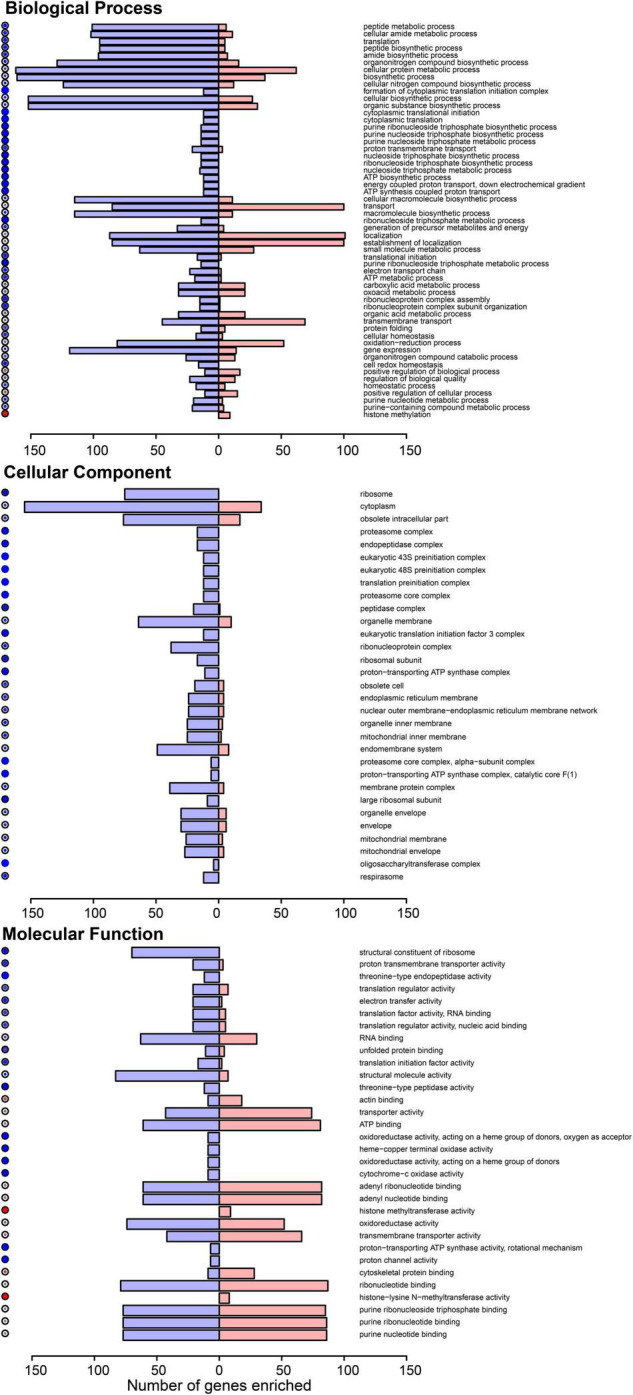
Gene ontology (GO) of the differentially expressed genes in *A. aegypti* ovaries transcriptomes 3 days after DENV-2 blood feeding vs. mock blood feeding. The color dots to the left of each category shows the dominating proportion of genes either downregulated (blue) or upregulated (red) within the GO category.

To explore the functional annotation trends more broadly beyond the 12 validated DEGs, we subjected all the significant DEGs (FDR < 0.05) to a Gene Ontology (GO) analysis, which was aided by the *Drosophila* ortholog names. At Benjamini-Hochberg corrected *p*-values < 0.05, we identified enriched biological processes, molecular functions, and cellular components among the significant mosquito ovary DEGs impacted by DENV-2 blood meal (Figure 3). A trend of physiological and metabolic functions was also apparent in these GO terms, such as peptide metabolic and translation processes, as well as ribosome and translation activity functions.

The general overview of the GO results showed a trend of downregulated genes within the GO biological process in the mosquitoes’ ovaries after DENV-2 blood meal (Figure 3 upper panel). This lowering of the biological process may be related to a decreased number of eggs observed in the first gonadotrophic cycle of the infected mosquitoes.

### Specific Differential Gene Expression Changes Validated in *Aedes aegypti* Ovaries During DENV-2 Infection

**FIGURE 4:**
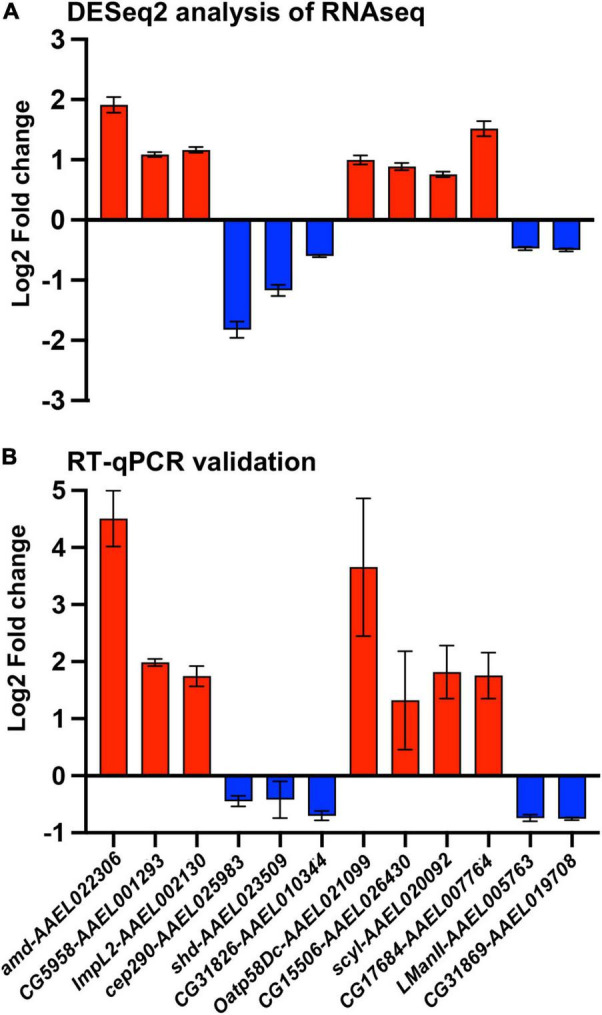
Validation of *A. aegypti* ovary genes differentially expressed between DENV-2 and mock infected mosquitoes. **(A)** Fold change of 12 differentially expressed genes from a DESeq2 analysis. **(B)** Validation of the 12 selected genes by RT-qPCR from mosquito ovaries 3 days post mock or DENV-2 infectious blood meal. The average fold change from two independent biological replicates, repeated at least three times and normalized against *A. aegypti* actin gene. Red colored bars represent upregulated genes, and blue colored bars represent downregulated genes.

From the gene expression results shown by the heatmap, we selected 12 DEGs for validation by qRT-PCR (Figure 4A). We chose 12 genes to have a better representation of the transcriptome and focus on a search for virally regulated genes. We performed two independent biological replicates of groups of mosquitoes that were fed uninfected blood (mock) vs. a DENV2-infected blood meal. At 3 days post blood feeding, we dissected the ovaries and tested the female carcasses to confirm DENV-2 infection. We used pools of DENV-2 positive ovaries for the RT-qPCR validation assay. As anticipated, all 12 DEGs we tested exhibited similar directional gene expression changes between the RNAseq experiment and the RT-qPCR experiment. Interestingly, two DEGs (amd-AAEL022306 and Oatp58Dc-AAEL021099) after qRT-PCR assay were confirmed to be most upregulated, with over a 3-fold increase, among the 12 selected genes (Figure 4B). However, these two genes were the best candidates for further investigation.

We decided to compare the gene expression of these two genes (*amd*-AAEL022306 and *Oatp58Dc*-AAEL021099) with that of an *A. aegypti* eggshell organization factor 1 (EOF1) (Isoe et al., 2019). The EOF1 was recently identified and plays a role in the formation and melanization of the eggshell. Our results using DENV-2 blood-fed females and mock-fed females, 3 days after feedings, showed no differences in the DENV-2-infected mosquitoes compared to the mock-infected ones (Supplementary Figure 2).

### Specific DEGs From the *Aedes aegypti* Ovary Transcriptome During DENV-2 Infection Also Display Responses in DENV-2 Infection of the Aag2 Cell Line

**FIGURE 5:**
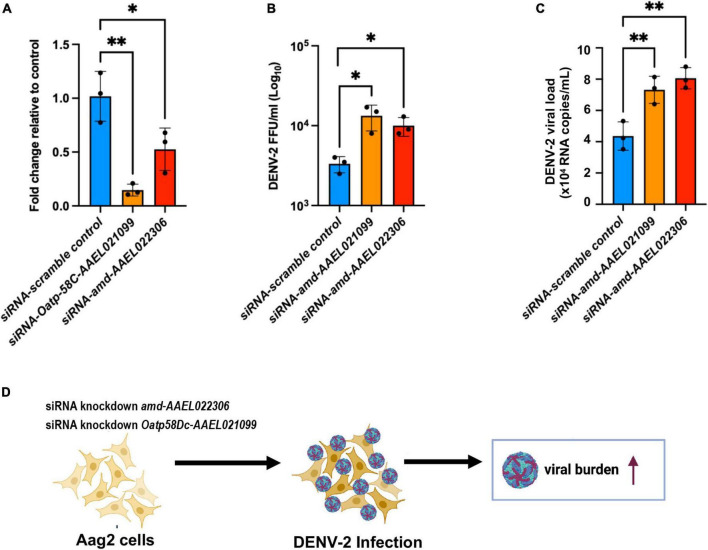
Silencing of genes *Oatp58C-AAEL021099* and *amd-AAEL022306* in *A. aegypti* Aag2 cell line after DENV-2 infection enhance viral infection. **(A)** Efficiency of gene knockdown in Aag2 cells measured at 3 days post transfection. Data were analyzed by one-way ANOVA with Šídák’s multiple comparisons test: *p < 0.05, ^**^p < 0.005. **(B)** Viral titer obtained by ffa, from Aag2 cell supernatants collected at 24 h post infection. Statistical significance of differences *p < 0.05, ^**^p < 0.001 in unpaired Student’s *t*-test. **(C)** RNA copies determined by RT-qPCR, using 10-fold dilution of DENV-2 standard RNA. Statistical significance of differences ^**^p < *0.001* in unpaired Student’s *t*-test. **(D)** Schematic of the experiment design summarizing a potential link between two mosquito genes *amd-AAEL022306* and *Oatp58C-AAEL021099* in response to DENV-2 infection.

We hypothesize that DEGs from the *A. aegypti* ovary transcriptome may regulate host responses to DENV-2 infection and modulate fecundity. Using an *in vitro* approach, we chose to investigate the roles of the most upregulated genes from the RT-qPCR validation assays using the Aag2 cell line. This cell line has been shown to recapitulate the immune responses found in adult mosquitoes (Zhang et al., 2017; Li M. J. et al., 2020; Rosendo Machado et al., 2021). Aag2 cells have been used to experimentally validate mosquito immune pathways (Russell et al., 2021) and to elucidate the mechanisms of antimicrobial peptides (AMP) (Zhang et al., 2017). The advantage of using Aag2 cells to study antiviral immunity genes is that they are much simpler to infect and perform small interfering RNA (siRNA) gene knockdowns than *in vivo* methods. In our Aag2 cell assays, we observed the ideal peak knockdown at 3 days post siRNA transfection of *Oatp58Dc-AAEL021099* and *amd-AAEL022* in Aag2 cells measured by RT-qPCR (Figure 5A). Following 3 days after knockdown, we infected the cells with DENV-2 at an MOI of 1. At 24 h post infection (24 hpi), cell culture supernatant was collected, and the viral titers were determined by ffa (Figure 5B). We also checked the viral loads by RT-qPCR (Figure 5C). We observed an increase in DENV-2 RNA copies in Aag2 cells after siRNA-mediated knockdown of either *Oatp58Dc-AAEL021099* or *amd-AAEL022306* compared to cells with the control scrambled siRNA, indicating a potential role of these genes in the mosquito antiviral response (Figure 5D). The same phenotype was observed by Ramirez and Dimopoulos (2010) when silencing MyD88 in laboratory strains of *A. aegypti*, DENV titers were increased in the mosquito’s midgut.

## Discussion

In this study, we observed that DENV-2 has an impact on the first gonadotrophic cycle of *A. aegypti*. Our data show that infected females lay significantly less number of eggs compared with females that were fed uninfected blood at the first gonadotrophic cycle. Although our data are discordant with a previous report by Sylvestre et al. (2013) where the authors observed a reduction in fecundity observable only at the third and subsequent gonadotrophic cycles, many factors could be affecting mosquito response to infection in laboratory settings, including specific mosquito strain, viral titer, feeding regimens, and others. Other reports suggest a similar reduction in fecundity starting in the first gonadotrophic cycle in the case of infection of *A. aegypti* with alphaviruses such as Mayaro virus (Alto et al., 2020), CHIKV virus (Sirisena et al., 2018), and with flaviviruses such as ZIKV (Petersen et al., 2018). There is not much attention or in-depth studies showing details on the impact of DENV and other members of the Flavivirus genus on the fecundity and fertility of *Aedes* mosquitoes. This is extremely important considering that mosquitoes, males and females, and the eggs are potential natural viral reservoirs (Sánchez-Vargas et al., 2018). Virally infected mosquitoes in nature constitute a powerful source of supporting viruses during intra-epidemic periods and a huge burden on public health (Chung et al., 1998). Studies in *Anopheles* mosquitoes show stimulation of the mosquito immune system by a pathogen and a direct cost within their reproduction and life span (Ahmed and Hurd, 2006; Araujo et al., 2011). For example, in *Anopheles gambiae*, authors used lipopolysaccharide (LPS) to generate a strong immune response that resulted in the upregulation of several immune genes, proposing that immune responses affect egg production *via* induction of apoptosis in the ovarian follicular cells, and this may be one of the mechanisms that lead to a reduction in egg production (Ahmed and Hurd, 2006). A few studies in *Aedes* spp. showed a viral impact on egg laying. CHIKV infected *A. aegypti*, for example, directly impacted the fitness of the mosquitoes by a low survival rate and a decreased number of eggs laid (Sirisena et al., 2018). Moreover, a recent study showed that infection with DENV changes the preference of where to lay their eggs by modifying the expression of selected genes associated with the olfactory learning processes, suggesting that DENV infection may not only impact the number of eggs laid but also the choice of where to deposit them (Gaburro et al., 2018).

Previous studies demonstrated that mosquito immune responses are modulated by blood feeding and that these changes continue through 3 days after ingesting an infectious blood meal when the females are ready to lay their eggs. Blood alone, without the presence of any pathogen, activates and upregulates immune-related genes (Giraldo-Calderón et al., 2020). In a recent study, the generation of GCTL-3^–/–^*Aedes aegypti* by CRISPR/Cas9 resulted in a reduction in DENV-2 infection, upregulation of Toll, IMD, and JAK/STAT immune pathways, a reduction in life span, and a reduction in egg production (Li H. H. et al., 2020). In DENV-resistant mosquitoes, the activation of the JAK/STAT pathway results in a fitness cost by decreased egg production by *Aedes* mosquitoes (Jupatanakul et al., 2017).

The extensive trend of metabolic and physiological functions among this set of ovarian DEGs in response to DENV represents an overlooked area of arbovirology since innate immune pathway genes were absent from our DEG analysis. For instance, the gene *amd*-AAEL022306 is predicted to participate in the decarboxylation and deamination of L-dopa to 3,4-dihydroxylphenylacetaldehyde (DHPAA). DHPAA is a highly toxic component since its aldehyde group readily reacts with the primary amino groups of proteins, leading to protein crosslinking and inactivation (Vavricka et al., 2011). The fact that silencing *amd*-AAEL022306 increases DHPAA following DENV infection suggests that it may participate in the inactivation of viral particles.

Similarly, the solute carrier organic anion transporter family member *Oatp58Dc*-AAEL021099 was also found to be upregulated in the ovaries of infected mosquitoes. The predicted biological process of this protein is ion/transmembrane transport. Interestingly, the silencing of this gene resulted in an increase in DENV replication in Aag2 cells. In *Drosophila*, *Oatp58Dc* is highly expressed in the Malpighian tubules, and it is associated with anoxia, which is linked with an increase in oxidative stress (Campbell et al., 2019). However, Aag2 cells are known as immune-responsive cells (Fallon and Sun, 2001) and have been validated as a reliable model to study *Aedes* immune responses against pathogens (Barletta et al., 2012). More experiments are needed to evaluate the effect of upregulation of *Oatp58Dc-AAEL021099* and *amd-AAEL022306* on immunomodulatory responses *in vivo*. These data suggest that the two genes identified in our mosquito ovary transcriptome may not be involved in the formation and melanization of the eggshell. In contrast, the viral upregulation of these two genes displayed characteristics of immune-related genes when compared to genes of the mosquito immune pathway.

Although our future goal will be to test the functional relevance of these DEGs in DENV-2 infected mosquitoes, we were able to assess *in vitro* the relevance of two of the DEGs in an Aag2 cell line, setting forth a platform for further investigation of the role that metabolic and physiologic gene may play in the mosquito immune response to arbovirus infection.

## Conclusion

By employing new strategies to assess mosquito transcriptomic changes during DENV-2 infection and combining gene annotation information from the *Drosophila* database, in this study, we present that DENV-2 elicited transcriptional signatures in the ovaries of *A. aegypti* and the impact of infection on the reproductive cycle, including the reduction in the number of eggs laid by infected females during the first gonadotrophic cycle. The differential gene expression data, shown in this study, are the first to outline the transcriptional signature of the ovary when the virus is not yet present, indicating that the ovaries are responding to the viral infection as early as 3 days after the female ingests an infectious blood meal. It is possible that DENV activates the immune system and triggers upregulation of ovarian genes, and this early response may directly interfere with the fecundity of the mosquitoes in first egg laying, as we observed a reduced number of eggs laid. Building upon these results, we also identified two DEGs that displayed an antiviral mechanism in response to viral infection in mosquito cell lines, indicating their putative cell-autonomous function in the antiviral response of the mosquito. Future investigations of how the early activation of mosquito host responses to DENV-2 affects the fecundity and reproductive fitness of *A. aegypti* are required and may provide new strategies for mosquito control to halt the spread of arboviruses.

## Data Availability Statement

The datasets presented in this study can be found in online repositories. The names of the repository/repositories and accession number(s) can be found below: https://www.ncbi.nlm.nih.gov/, SRR17125477; https://www.ncbi.nlm.nih.gov/, SRR17125482.

## Ethics Statement

The animal study was reviewed and approved by the Institutional Animal Care and Use Committee (IACUC). Approved protocol PROTO201900005.

## Author Contributions

FF-S, TC, and BL-R: conceptualization. FF-S, ZZ, EM, and AG: mosquito and virology assays. AB-C, AT, and MR-B: laboratory assistance. GD and NL: bioinformatic analyses and writing. RC: provide foundational and infrastructural support and oversight. FF-S: initial draft. FF-S and BL-R: final writing, review, and editing. All authors reviewed and provided comments on the text.

## Conflict of Interest

TC was employed by the Moderna Inc. The remaining authors declare that the research was conducted in the absence of any commercial or financial relationships that could be construed as a potential conflict of interest.

## Publisher’s Note

All claims expressed in this article are solely those of the authors and do not necessarily represent those of their affiliated organizations, or those of the publisher, the editors and the reviewers. Any product that may be evaluated in this article, or claim that may be made by its manufacturer, is not guaranteed or endorsed by the publisher.
