# Supplementary figures and images for: Dengue Virus-2 Infection Affects Fecundity and Elicits Specific Transcriptional Changes in the Ovaries of Aedes aegypti Mosquitoes
Source: Front Microbiol. 2022 Jun 23;13:886787. doi: 10.3389/fmicb.2022.886787 (PMC9260120; doi:10.3389/fmicb.2022.886787)

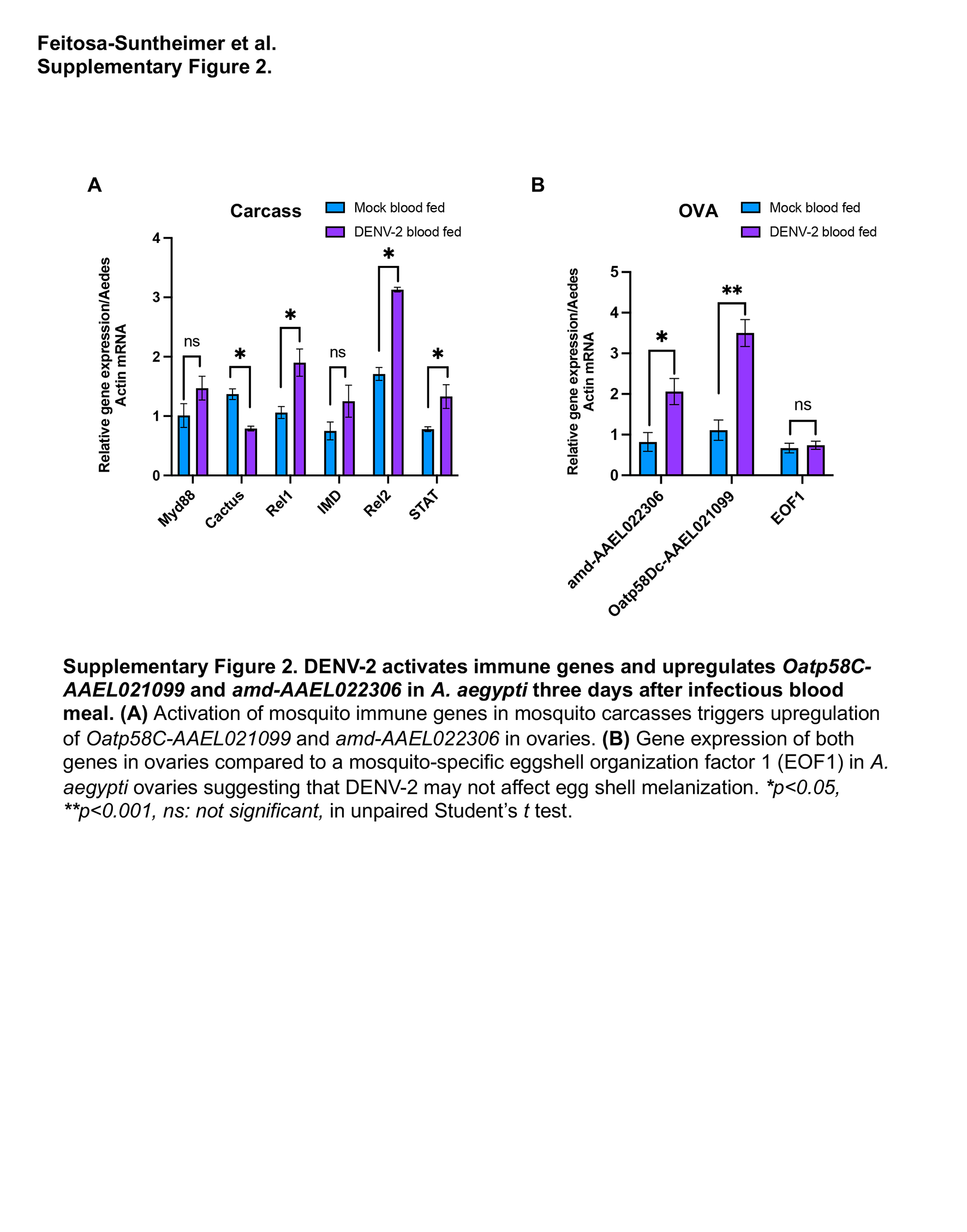

Supplement: Supplementary file 2 [file Image_2.tif]
