# Supplementary material for: Dengue Virus-2 Infection Affects Fecundity and Elicits Specific Transcriptional Changes in the Ovaries of Aedes aegypti Mosquitoes
Source: Front Microbiol. 2022 Jun 23;13:886787. doi: 10.3389/fmicb.2022.886787 (PMC9260120; doi:10.3389/fmicb.2022.886787)
Supplement: Supplementary file 3 [file Table_1.pdf]

Supplementary Table 1: Gene specific primers

primers used for RT-qPCR-validation of RNA-Seq		
**Gene ID-VectorBase**	**Forward primer (5'-3')**	**Reverse primer (5'-3')**
**AAEL001293**	GTGTGGGCTCTCTTCACTCC	TCCACGTATTTTCCCACGCA
**AAEL002130**	GTTCACGGCAGTGGTCAAAC	TGGTCGAAGCAAGAGCCATT
**AAEL010344**	TTGCCGACGACGAGAAGAAA	AGCGTCATCAACGTTCCAGT
**AAEL022306**	TGTGCTCCCATCTGTCGAAC	GTCAGGCCAGGGACAATGAA
**AAEL023509**	CCTCAGGTATCATGTCCCGC	AACAGTGTTCGCGATGTCCT
**AAEL025983**	CGGCAAACAACGAGAAGCAA	TACCGCGAAAGAGTGGTGTC
**AAEL005763**	ACCTCCTCCTGGATTCTGCT	AGTGCGGTAAGACGAACTCA
**AAEL007764**	GCTTATGTGAGCCCTCCCTC	CAGCGAACACCATGCTCGTA
**AAEL019708**	TGCGAAAAGAAGCACGGTTG	CTCCTTGGCAGTAGCACACA
**AAEL020092**	CCATCGACGATCTGGTTGAAGA	GCGTTGGTTGGCGTTCTTAT
**AAEL021099**	AGGACATGCCACTGACGAAG	CCCACGACACCGTACAGAAA
**AAEL026430**	ACCAAACAACAGCATCGCAC	GCAGGTTGTACGCTTGTTGG

primers used for siRNA in cells		
**Gene ID-VectorBase**	**Forward primer (5'-3')**	**Reverse primer (5'-3')**
**siRNA-AAEL022306**	GGCATTTAGTTGAAACGATAATAAA	TTTATTATCGTTTCAACTAAATGCCAT
**siRNA-AAEL021099**	GATGGAGAATCATTGAGGTACCTCA	TGAGGTACCTCAATGATTCTCCATCAT

primers used for immune genes		
**NCBI-Genbank**	**Forward primer (5'-3')**	**Reverse primer (5'-3')**
**MyD88: XM_001658585**	CGTGATTGGCGAGGGTTGTTTC	ATCCGCTCCAATGCTCGTTTCC
**CACTUS: XM_021848127.1**	TCTTGCGTTGAAGTGAGTGG	GACCCTCTGAAAGGGAAAGG
**REL1: AY748242.1**	GACTCGTCGGAGCTGAAATC	CGGTTTGTTCAGGTTGTTGA
**Cecropin (CecN): KF923689.1**	CGGCAAGAAATTGGAAAAAGTC	GAATCGATCATCCTAGGGCC
**Defensin (DefA): KX890447**	CTATCAGGCTGCCGTGGAG	CAATGAGCAGCACAAGCACTATC
**IMD: GFNA01096113.1**	TCGTCAAACTCGGTTTTCCT	TGGCGGAGTTGAAGGTAAAG
**REL2: AF498105.1**	TCTGTCGGCAGATGAAGTGA	GCACTGGAATGGAGATCTAAA
**STAT: XM_021839341.1**	CACACAAAAAGGACGAAGCA	TCCAGTTCCCCTAAAGCTCA

***Ae.aegypti* -ACTIN-XM_001655126.2**	GAACACCCAGTCCTGCTGACA	TGCGTCATCTTCTCACGGTTAG
**DENV-2-NGC strain**	CAGATCTCTGATGAATAACCAACG	CATTCCAAGTGAGAATCTCTTTGTCA
